# Nipah Virus in Lyle's Flying Foxes, Cambodia

**DOI:** 10.3201/eid1107.041350

**Published:** 2005-07

**Authors:** Jean-Marc Reynes, Dorian Counor, Sivuth Ong, Caroline Faure, Vansay Seng, Sophie Molia, Joe Walston, Marie Claude Georges-Courbot, Vincent Deubel, Jean-Louis Sarthou

**Affiliations:** * Institut Pasteur du Cambodge, Phnom Penh, Cambodia;; † Institut Pasteur, Lyon, France;; ‡ Wildlife Conservation Society, Phnom Penh, Cambodia

**Keywords:** Cambodia, Nipah virus, Chiroptera, Pteropus

## Abstract

We conducted a survey in Cambodia in 2000 on henipavirus infection among several bat species, including flying foxes, and persons exposed to these animals. Among 1,072 bat serum samples tested by enzyme-linked immunosorbent assay, antibodies reactive to Nipah virus (NiV) antigen were detected only in *Pteropus lylei* species; *Cynopterus sphinx*, *Hipposideros larvatus*, *Scotophilus kuhlii*, *Chaerephon plicata*, *Taphozous melanopogon*, and *T. theobaldi* species were negative. Seroneutralization applied on a subset of 156 serum samples confirmed these results. None of the 8 human serum samples was NiV seropositive with the seroneutralization test. One virus isolate exhibiting cytopathic effect with syncytia was obtained from 769 urine samples collected at roosts of *P. lylei* specimens. Partial molecular characterization of this isolate demonstrated that it was closely related to NiV. These results strengthen the hypothesis that flying foxes could be the natural host of NiV. Surveillance of human cases should be implemented.

The new genus *Henipavirus* contains 2 species, Hendra virus (HeV) and Nipah virus (NiV), in the subfamily of the *Paramyxovirinae*, which also includes *Respirovirus*, *Rubulavirus*, *Morbillivirus*, and *Avulavirus* genera (*1*). HeV and NiV have emerged within the last 10 years and have been shown to be highly pathogenic in animals and humans. HeV was identified in 1994 in Brisbane during an outbreak of acute respiratory syndrome in 21 horses, of which 14 died. Two humans were also affected with the same syndrome, and 1 died (*2**,**3*). A second outbreak that occurred 1 year later ≈1,000 km north of Brisbane caused the death of 2 horses and their owner. Severe encephalitis followed by mild meningitis affected the owner 1 year after his exposure (*4*). NiV was discovered in Malaysia during a major outbreak of acute respiratory syndrome in pigs in 1998; it caused severe acute encephalitis among >283 pig farmers (with 109 deaths) and among 35 abattoir workers in Singapore (*5**,**6*). In 2001, 2003, and 2004, this virus emerged in Bangladesh; 4 outbreaks were reported with high death rates (32%–75%) (*7*). Person-to-person transmission was suspected during the last outbreak (*8*), which raised concerns about human infection from an epizootic or deliberate release of this highly virulent virus. Serologic and virologic investigations suggested that these 2 viruses shared *Pteropus* bats or flying foxes as natural host reservoirs (*9**–**12*). Furthermore, 2 other paramyxoviruses belonging to the genus *Rubulavirus* have been associated with flying foxes: Menangle virus, isolated in 1997 from pigs in Australia (*13*), and Tioman virus, isolated in 1999 from fruit bats in Malaysia (*14*).

Distribution of the henipaviruses is restricted so far to Australia, Malaysia, and Bangladesh. The distribution of flying foxes (≈58 species), considered as natural hosts of these viruses, extends from the east coast of Africa across south and Southeast Asia, east to the Philippines and Pacific islands, and south to Australia (*15*). It could be conjectured that henipavirus or similar viruses occur in flying foxes elsewhere and could emerge as a human pathogen. Three species belonging to the genus *Pteropus* (*Pteropus lylei*, *P. hypomelanus*, and *P. vampyrus*) have been identified in Cambodia (*15*), and antibodies to NiV-like virus have recently been detected in Lyle's Flying Fox (*P. lylei*) (*16*).

We conducted a survey in Cambodia, initiated in 2000, on henipavirus infection among several bat species, especially flying foxes, and persons exposed to these animals. Here, we confirm the presence of NiV antibodies and report NiV isolation and characterization from Lyle's bats in Cambodia.

## Materials and Methods

### Sample Collection

A total of 1,303 bats were sampled from 35 locations in 9 Cambodian provinces, from September 2000 to May 2001. Of these, 467 came from restaurants in Phnom Penh and belonged to the species *P. lylei*. Blood samples were collected at the restaurants when the animals were killed for meat. The other 836 animals were captured in 9 provinces at roosts by hand and with hand nets, or along flyways by night with mist nets or harp traps. Anesthetized captured animals were euthanized by cardiac blood puncture. These animals belonged to 16 species representing 6 of the 7 bat families known from Cambodia (*17*). Serum samples from 1,072 animals were taken for serologic investigation.

**Figure 1:**
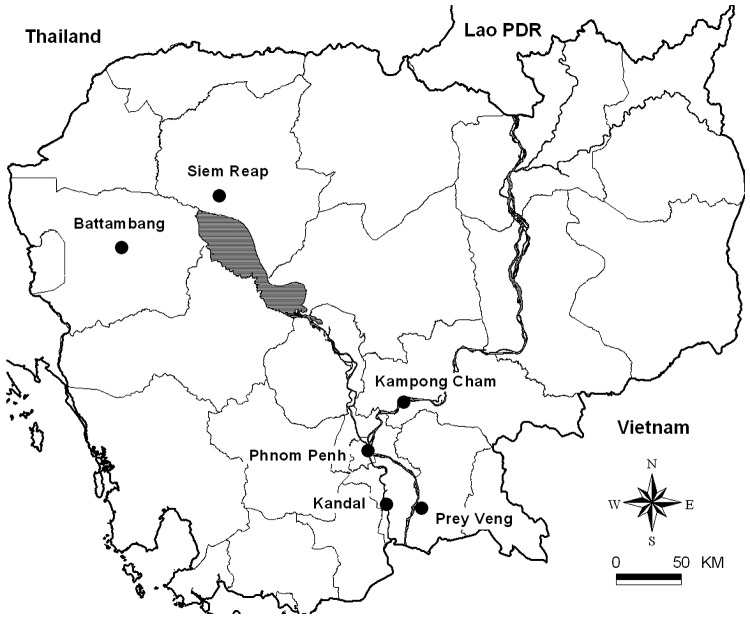
Bat urine sampling site. Cambodia, June 2003–July 2004.

Under 6 roosts of flying foxes (where individual bats from the 2 species *P. lylei* and *P. vampyrus* could live together), urine samples were collected from June 2003 to August 2004 on plastic sheets, following a published procedure (*18*). The roosts, of 400 to 600 flying foxes each, were located in Phnom Penh, Battambang, Kampong Cham, Kandal, Prey Veng, and Siem Reap Provinces (Figure 1). The roosts were visited 4 times, when the animals were present (the roosts are deserted from December to May). No roost of Variable Flying Foxes (*P. hypomelanus*) could be located. A total of 769 urine samples stored at –80°C were available for virologic investigation.

Serum samples were obtained in January 2001 from 8 persons who gave their oral consent and who were exposed to NiV-seropositive Lyle's Flying Fox bats. Four men worked in restaurants where bats are eaten and handled the animals; 4 women worked at the same place and slaughtered and cooked the bats.

### Serologic Tests

Bat sera were first screened for antibodies against NiV by enzyme-linked immunosorbent assay (ELISA), as described (*19*). Antigens were prepared in the biosafety level Laboratory Jean Merieux in Lyon from Vero E6 cells infected with NiV and inactivated by γ irradiation as described (*20*). Sera were diluted 1:100. Peroxidase-labeled protein A/G (Pierce, Rockford, IL, USA) was used as conjugate. Three negative control serum samples and 1 positive control sample were included in each run.

Seroneutralization tests were carried out under biosafety level 4 conditions. Serum samples were heated for 30 min at 56°C and then were titrated with 3 dilutions (1:10; 1:20, and 1:40) in a 96-well microtiter plate (4 wells per dilution). Equal volume of NiV (100 50% tissue culture infective dose [TCID_50_] in 50 μL) was added to all sera, and the plate was incubated for 1 h at 37°C. Vero E6 cells (5 × 10^4^ in 100 μL) were added to all wells, and the plates were incubated at 37°C for 4 days in a CO_2_ chamber at 37°C. Characteristic NiV cytopathic effect (CPE) showing large syncytia was observed under a microscope in each well. The number of virus-positive wells was confirmed after fixation in 10% formaldehyde for 1 h and amido-schwartz staining for 30 min. Positive (anti-NiV serum obtained from a convalescent-phase specimen) and negative sera were included for controls in each plate. Toxicity of the sera for Vero cells was observed on uninfected cells in the presence of 1:10 serum dilution. The neutralization titer of each sample was defined as the last dilution in which at least half of the monolayer was intact (TCID_50_).

### Virus Isolation and Identification

Subconfluent Vero E6 cells (ATCC CRL-1586) in flasks of 25 cm^2^ with filter caps were inoculated with 500 μL of viral transport medium containing the urine-impregnated cotton swab. The cell cultures were placed in a CO_2_ incubator at 37°C and examined daily for CPE such as formation of multinucleated giant cells. If a CPE was observed, supernatants and cells were collected separately and frozen at –80°C.

RNA was extracted from supernatants by using QIAMP viral RNA mini kit (Qiagen, Hilden, Germany) according to the manufacturer's procedure. Identification of paramyxoviruses or NiV were performed with reverse transcriptase–polymerase chain reaction (RT-PCR) with specific primers for the phosphoprotein (P) gene of *Paramyxoviridae* or specific primers for the nucleoprotein (N) gene of NiV (*21*).

### Molecular Characterization

To amplify and sequence the coding domains of the N and glycoprotein (G) genes, primers were designed by using Primer3 software (*22*), according to the published nucleotide sequences of NiV. Reverse transcription and amplification were conducted with these primers and RNA extracted from supernatants of positive isolates. The amplified products were observed after electrophoresis on a 1% agarose gel with ethidium bromide staining; then purified, amplified products were sequenced with the dye termination cycle sequencing technique (Genome Express Company, Meylan, France). Sequences sent by the company were verified and aligned by using ClustalW program version 1.83 (*23*). Phylogenetic analysis was conducted with PHYLIP sequence analysis package (version 3.6 alpha 3) (*24*).

## Results

### Serologic Testing

Results of ELISAs for antibodies reactive with NiV antigens in 1,072 bats serum samples are shown in the Table. A positive signal was identified only in *P. lylei*. Fifty (10.9%) of the 458 tested samples from this species were positive.

**Table:** Serum samples from 1,072 bats, Cambodia, reactive by enzyme-linked immunosorbent assay to Nipah virus, September 2000–May 2001

Species names	Negative	Positive
Frugivorous		
*Cynopterus brachyotis*	1	0
*Cynopterus sphinx*	68	0
*Macroglossus sobrinus*	1	0
*Pteropus lylei*	408	50
*Roussetus leschenaulti*	15	0
Insectivorous		0
*Chaerephon plicata*	153	0
*Hipposideros armiger*	1	0
*H. larvatus*	81	0
*H. pomona*	2	0
*Murina cyclotis*	1	0
*Rhinolophus acuminatus*	2	0
*R. luctus*	1	0
*Scotophilus kuhlii*	98	0
*Taphozous melanopogon*	69	0
*T. theobaldi*	121	0
Total	1,022	50

Forty-three of the 50 ELISA-positive bat serum samples were available for confirmation by serum neutralization test. One produced cell toxicity, 1 was negative (titer <1:10), and 41 were positive. Neutralization at dilutions of 1:10, 1:20, and ≥1:40 was found in 1, 11, and 29 serum specimens, respectively. A subset (n = 156) of the 1,022 ELISA-negative serum specimens were tested by seroneutralization; 5 produced cell toxicity. Six among 43 ELISA-negative serum samples from *P. lylei* were positive (4 with a titer = 1:10 and 2 with a titer = 1:20). The 108 ELISA-negative serum samples from other captured species, *Cynopterus sphinx* (n = 15), *Hipposideros larvatus* (n = 15), *S. kuhlii* (n = 15), *Chaerephon plicata* (n = 27), *Taphozous melanopogon* (n = 12), and *T. theobaldi* (n = 24), were negative. None of the human sera were NiV seropositive with the seroneutralization test.

### Virus Isolation and Molecular Characterization

Attempts to isolate viruses producing a CPE were positive for 2 samples among the 769 urine samples. These 2 samples, CSUR381 and CSUR382, were collected consecutively on the same morning (April 23, 2004) at the same roost of *P. lylei* specimens, located in a pagoda in the village of Bay Damran in Battambang Province. CPE showing syncytia was detected 6 days after injection into Vero E6 cells.

RT-PCR performed on RNA extracted from infected cell supernatants using specific primers for the P gene of *Paramyxoviridae*, as well as specific primers for the N gene of NiV, produced PCR products of the expected size (138 bp and 228 bp, respectively). When the 2 partial N region products from the 2 Cambodian strains were sequenced, 2 identical nucleotide sequences were found that shared 97.4% homology to that of the NiV strain isolated in Malaysia, a finding that suggests that the Cambodian isolates belonged to the NiV species.

**Figure A1:**
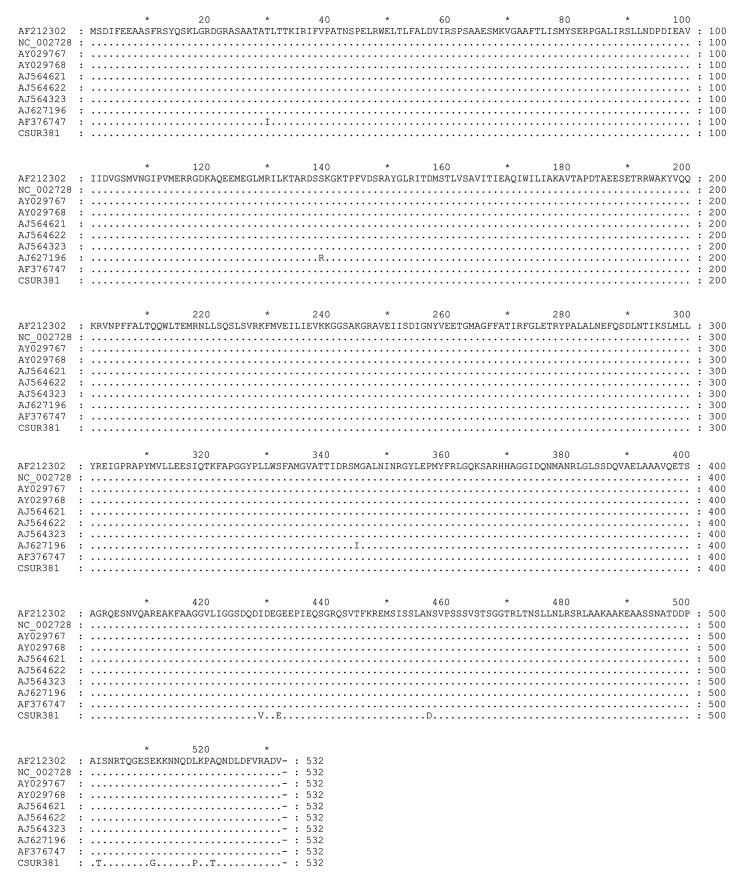
Comparison of the N proteins of Nipah virus (NiV) strains from Malaysia and Cambodia. The alignment shows the predicted amino acid sequence of the N protein of the Malaysian human strain (GenBank accession no. AF212302) and indicates positions that differ in the N proteins. Numbers correspond to amino acid positions in the NiV N protein; stars marks lengths of 10 amino acids; dots indicate identical amino acids.

**Figure A2:**
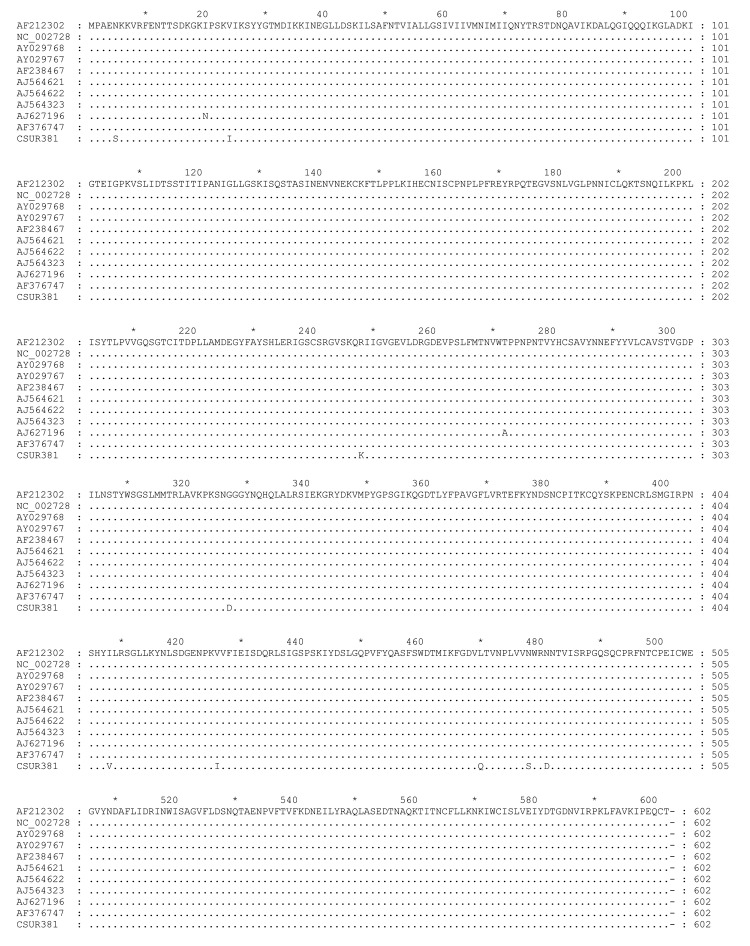
Comparison of the G proteins of Nipah virus (NiV) strains from Malaysia and Cambodia. The alignment shows the predicted amino acid sequence of the G protein of the Malaysian human strain (GenBank accession no. AF212302) and indicates positions that differ in the N proteins. Numbers correspond to amino acid positions in the NiV N protein; stars marks lengths of 10 amino acids; dots indicate identical amino acids.

Further molecular characterization was achieved when the N and G genes of CSUR381 and CSUR382 isolates were amplified and sequenced. The nucleotide sequences of the 2 isolates were identical. The N sequence of CSUR381 was compared with the N sequences of the Malaysian NiV strains available in November 2004 in GenBank (AF212302, NC-0027281 derived from AF212303, AY029768, and AY029767 from human isolates; AJ564621, AJ564622, AJ564623, and AJ627196 from pig isolates; and AF376747 from a *P. hypomelanus* isolate). We observed that the Cambodian N nucleotide sequence shared 98% identity with the Malaysian AF21232 N sequence (32 nucleotides among 1,599 were divergent). The identity was 98.7% (525/532) for the N amino acid sequences (Figure A1). All the changes of the 532 amino acid (aa) N protein occurred at the carboxyl terminus, with the following mutations: I429V, G432E, N457D, I502T, E511G, L518P, and A521T. The G sequence of the Cambodian CSUR381 strain was also compared to the available G sequences of the Malaysian NiV isolates. The percentage of nucleotide homology was 98.2% (32 nucleotide changes among 1,809), and the percentage of amino acid homology was 98.5% (593/602). Amino acid changes were N5S, V24I, R248K, G327D, I408V, V426I, L470Q, N478S, and N481D (Figure A2). These results confirmed that the Cambodian isolates are closely related to the other NiV isolates.

**Figure 2:**
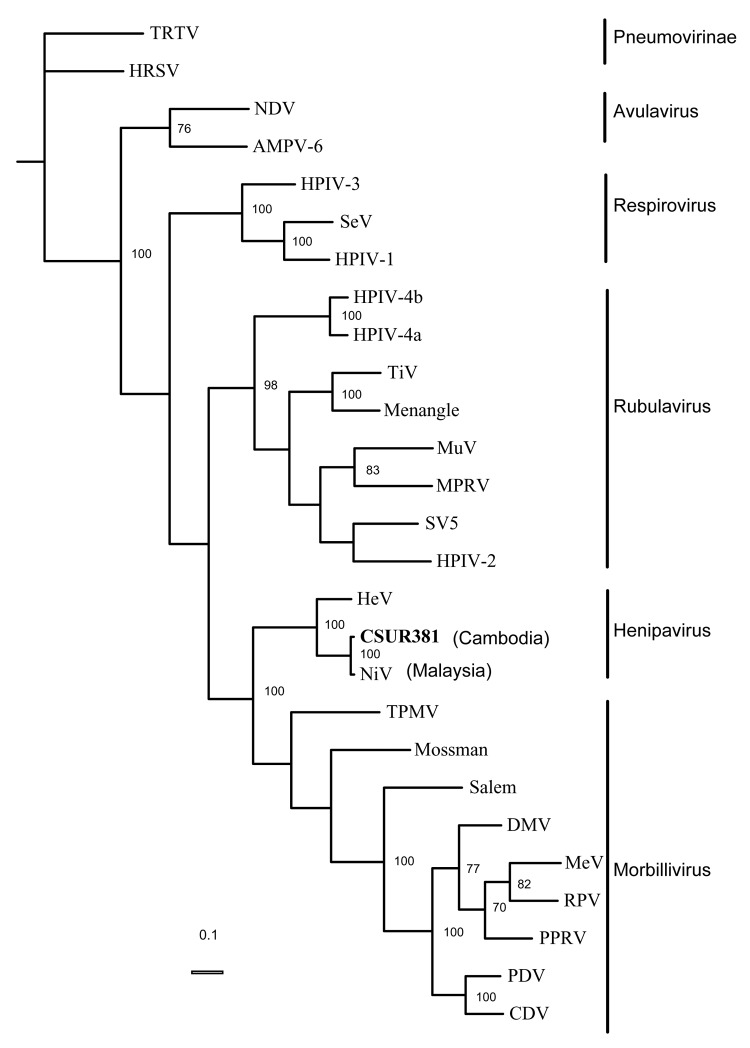
Phylogenetic analysis of the 1,599 nucleotides of the N gene coding domain sequence from the Nipah virus Cambodian isolate, members of the subfamily *Paramyxovirinae*, and 2 species of the subfamily *Pneumovirinae* used as outgroups. GenBank accessions numbers used are as follows: APMV-6: Avian paramyxovirus 6, AY029299; CDV: Canine distemper virus, AF014953; DMV, Dolphin distemper virus, X75961; HeV: Hendra virus, AF017149; HPIV-1: Human parainfluenza virus 1, D011070; HPIV-2: Human parainfluenza virus 2, M55320; HPIV-3: Human parainfluenza virus 3, D10025; HPIV4a: Parainfluenza virus type 4A, M32982; HPIV4b: Parainfluenza virus type 4B, M32983; HRSV: Human respiratory syncytial virus, X00001; MeV: Measles virus, K01711; MPRV: Mapuera virus, X85128; Menangle: Menangle virus, AF326114; Mossman: Mossman virus, AY286409; MuV: Mumps virus, D86172; NDV: Newcastle disease virus, AF064091; NiV: Nipah virus, AF212302; PDV: Phocid distemper virus, X75717; PPRV, Peste des Petits ruminants virus, X74443; RPV: Rinder pest virus, X68311; Salem: Salem virus, AF237881; SeV: Sendai virus, X00087; SV5: Simian virus 5, M81442; TiV: Tioman virus, AF298895; TPMV: Tupaia paramyxovirus, AF079780; TRTV: Turkey Rhinotracheitis virus, AY640317. Significant bootstrap values (≥70%) are indicated. The phylogram was generated by parsimony method and analyzing 100 bootstrap replicates.

Phylogenetic analysis using parsimony method (*24*) and different N sequences of viruses of the *Paramyxovirinae* subfamily confirmed that the Cambodian isolates and the Malaysian NiV isolates were significantly similar and that the Cambodian strains probably belong to the NiV species (Figure 2). Analyses performed by using other methods (neighbor joining and maximum likelihood) or using the G sequences reached the same conclusion (data not shown). The N and G nucleotide sequences of the isolate CSUR381 were deposited in GenBank (accession nos. AY858110 and AY858111).

## Discussion

Our serologic study confirms the presence in Cambodia of antibodies to a NiV-like virus among Lyle's Flying Foxes (*P. lylei*), as reported by Olson et al. in 2002 (*16*). We could not detect antibodies against NiV among bats belonging to other genera, including insectivorous bats. Some Malaysian specimens of 2 frugivorous species (*Cynopterus brachyotis* and *Eonycteris spelaea*) and 1 insectivorous species (*S. kuhlii)* have been found carrying neutralizing antibodies to NiV in Malaysia. These specimens were collected soon after an outbreak, when the virus was expected to circulate with high prevalence (*10*). Data on bat infection at sites of outbreaks in Bangladesh are limited, although antibodies against NiV have been detected in the Indian Flying Fox, *P. giganteus*, a species possibly conspecific with *P. vampyrus* (*7*). Although our sample is not representative of the bat population in Cambodia, our results strengthen the hypothesis that flying foxes could be the natural host of NiV. The results of both ELISA and neutralization tests used on this convenient panel of sera gave a relative sensitivity of 87% (41/47) and specificity of 99% (145/146). These findings are in accordance with the ELISA performance observed in other studies (*25*).

This description of NiV is the first in Cambodia. NiV has been isolated in 2 other countries. The NiV isolate obtained from *P. lylei* is the first isolate obtained from a *Pteropus* species different from *P. hypomelanus*, the first NiV-infected species in Malaysia. Investigation in Bangladesh, where the NiV outbreak occurred, did not detect any virus strains among bats; antibodies to Nipah-like virus were detected among specimens belonging to the species *P. giganteus* (*7*). Our results indicate that further henipavirus-related infection can be expected within the area of distribution of flying foxes and that NiV could emerge within this wide area.

Identification of the isolate was performed by molecular characterization on the N and G coding domains located at the 2 ends of structural proteins genes. We observed a higher amino acid diversity at the carboxyl terminus of the N protein between the Malaysian and Cambodian NiV isolates. The N central domain (from aa 171 to aa 383) of the members of the *Paramyxovirinae* family is the most conserved region, which seems to be involved in its interactions with other functional proteins, such as the phosphoprotein (P) and the polymerase (L). Moreover the N proteins of paramyxoviruses possess 3 highly conserved regions (*26*). The first region (QXW(I,V)XXXK(A,C)XT, X representing any amino acid) is located between aa 171 and aa 181, the second region (FXXT(I,L)(R,K)u(G,A)(L,I,V)XT, where u represents an aromatic amino acid) from aa 267 to aa 277, and the third region (FXXXXYPXXuSuAMG) from aa 322 to aa 336. All 3 regions were conserved in the sequence of the Cambodian NiV strain. Conversely, the C-terminal part of N (from aa 384 to aa 532) overlaps antigenic epitopes and is the most variable domain (*26*). However, no change occurred in the 29 aa C terminal region (aa 468-496) involved in the binding to the P protein (*27*). Lastly, the N481D change observed in the Cambodian NiV G protein induced the disappearance of 1 of the 8 N-linked glycosylation sites of the protein (*28*).

Only 2 NiV isolates were obtained from 6 roosts in 6 provinces that were investigated. This finding is not unusual with these pathogens and bats: no NiV strain was isolated from Variable Flying Foxes (*P. hypomelanus*) and Large Flying Foxes (*P. vampyrus*) during the 1999 Malaysian outbreak (*11*). Two NiV isolates were obtained in the postoutbreak period (August 1999–June 2000) from 263 urine samples of Variable Flying Fox bats (*12*) and, to our knowledge, no isolate from bats in Bangladesh has been reported. Furthermore, only 3 isolates of HeV were obtained from 652 bats tissue samples collected around the epidemic sites in Australia (*9*). The presence of antibodies in roosts from Kompong Cham Province suggests that the virus could be widely present in the country. However, the low rate of NiV recovery from *P. lylei*, when compared to the high number of NiV antibody–positive bats (10.9%), suggests that the virus may not be sustained lifelong in animals or that it may remain at low titers and occasionally emerges and is released in biologic fluids. The 8 persons exposed to Lyle's Flying Fox bats, some of which were NiV-seropositive, were NiV-seronegative. Thus, the animals may not have been carrying the virus during their captivity.

This is the first time an NiV has been isolated in a country where no outbreak has been reported. This situation requires strategies to manage this pathogen and to prevent an outbreak. Recommendations should be made to the population in areas where flying foxes' roosts are identified to minimize exposure to them. A national hospital-based surveillance of acute encephalitis should be implemented; in the upcoming months, such surveillance will be instituted in the area where the virus was isolated. Surveillance of respiratory syndrome among pigs has to be estimated, as intensive pig farming is not the rule in the area. The potential of the Cambodian NiV isolate to cause fatal encephalitis in hamsters will also be investigated (*29*). Lastly, ecologic studies, including the dynamics of flying fox populations and their relationships with NiV, should be considered for a better understanding of its transmission and maintenance among these populations.
